# Optimizing Recovery in Cardiac Surgery: A Narrative Review of Enhanced Recovery After Surgery Protocols and Clinical Outcomes

**DOI:** 10.3390/medsci13030128

**Published:** 2025-08-14

**Authors:** Arzina Jaffer, Kayleigh Yang, Alisha Ebrahim, Amy N. Brown, Ryaan EL-Andari, Aleksander Dokollari, Alex J. Gregory, Corey Adams, William D. T. Kent, Ali Fatehi Hassanabad

**Affiliations:** 1 Department of Anesthesiology and Pain Medicine, University of Alberta, Edmonton, AB T6G 2X8, Canada; 2 Department of Anesthesiology and Pain Medicine, University of Calgary, Calgary, AB T2N 2T9, Canada; 3 Department of Pediatrics, Faculty of Medicine, University of Ottawa, Ottawa, ON K1H 8L1, Canada; 4 Section of Cardiac Surgery, Department of Cardiac Sciences, Libin Cardiovascular Institute, University of Calgary, Calgary, AB T2N 2T9, Canada; 5 Division of Cardiac Surgery, University of Alberta, Edmonton, AB T2N 2T9, Canada; 6 Section of Cardiac Surgery, Department of Surgery, St Boniface Hospital, University of Manitoba, Winnipeg, MB R2H 2A6, Canada

**Keywords:** ERAS, cardiac surgery, patient outcomes

## Abstract

Enhanced Recovery After Surgery (ERAS) is an evidence-based, holistic perioperative recovery protocol intended to improve patient outcomes and decrease postoperative complication rates. While ERAS protocols were first introduced in 1997, specific guidelines for cardiac surgery were not established until 2019. Although the core principles of ERAS remain constant across surgical disciplines, ERAS guidelines for cardiac surgery have remained relatively understudied, likely due to the unique complexities posed by cardiac procedures. Within this comprehensive narrative review, we aimed to explore the current guidelines and evidence for ERAS in both cardiac and non-cardiac surgeries. In non-cardiac surgeries, ERAS has been shown to improve various outcomes, including ICU length of stay, patient satisfaction, and pain management. ERAS for cardiac surgery has also shown encouraging results, including shorter ICU and hospital stays, reduced postoperative opioid use, and faster recovery times. However, there is limited consensus across studies, particularly regarding its impact on morbidity and mortality, with mixed results reported. Furthermore, the limited data on the efficacy of ERAS in minimally invasive cardiac surgeries makes it difficult to establish well-supported guidelines for these procedures. Despite its limitations, the overall outcomes of ERAS for cardiac surgery remain promising. As our understanding and application of ERAS in cardiac surgery continue to evolve, these protocols have the potential to redefine cardiac surgical care standards.

## 1. Introduction

Enhanced Recovery After Surgery (ERAS) is a multimodal perioperative protocol that has transformed the care and management of surgical patients [1]. This approach aims to improve surgical outcomes and reduce postoperative complications by applying standardized, evidence-based practices throughout the perioperative period [1]. ERAS has traditionally focused on comprehensive preoperative patient education and optimization, prevention of surgical site infections, multimodal and opioid-sparing analgesia, early mobilization, and enhanced nutrition [1]. As ERAS protocols continue to advance, they hold the potential to improve the overall surgical experience for both patients and healthcare professionals [1].

While the key features of ERAS remain consistent across various surgical disciplines, ERAS protocols in cardiac surgery have been relatively newer and less studied [2]. The unique challenges, inherent complexities, and heterogeneity of cardiac procedures appear to be key hurdles to effective ERAS implementation in this area [2]. Overall, modern cardiac surgery has consistently demonstrated a high level of safety with minimal surgical complication rates, reflecting the inherent reliability and precision of established surgical techniques [3]. As a result, 80% of preventable morbidity and mortality associated with cardiac surgery now originates outside the operating room, highlighting the importance of further optimizing patient care during the perioperative period [3].

Despite ERAS protocols originating in 1997, the principles of ERAS in cardiac surgery only recently gained popularity following the release of ERAS Cardiac Surgery Guidelines in 2019 [4]. The gradual adoption of ERAS practices in cardiac surgery has been further complicated by the heterogeneous nature of the patient population undergoing various forms of cardiac interventions [4]. Due to the intricacies inherent to this field, it becomes important to adopt a nuanced approach and tailor ERAS protocols to address the diverse needs of cardiac patients [4]. Indeed, preliminary studies focused on ERAS and cardiac surgery have identified decreased time to extubation, shorter hospital and ICU lengths of stay, fewer postoperative complications, and fewer hospital readmissions with ERAS use [2,5].

Within this comprehensive narrative review, we aim to explore the existing body of evidence concerning ERAS protocols in both cardiac and non-cardiac surgery. We particularly focus on diverse surgical techniques and patient demographics to underscore the significance of identifying areas in need of further study.

## 2. Methods

This is a comprehensive narrative review. The following mesh and key terms were used: “early recovery after surgery”; “early recovery after cardiac surgery”; “ERAS”; “ERACS”; “cardiac surgery”; “guidelines”; “protocols”; “ERAS protocol”; and “ERACS protocols”. The search was completed on MedLine and PubMed. All article titles and abstracts were reviewed by A.J., K.Y., A.E., and A.F.H. All published papers retrieved on those search engines were considered with no time-frame limitations set.

## 3. ERAS in Non-Cardiac Surgery

### 3.1. History and Evolution

The history and evolution of ERAS in non-cardiac surgery can be traced to the pioneering studies of Kehlet in the 1990s [1,6]. These studies laid the foundation for ERAS principles by highlighting the importance of mitigating the surgical stress response and optimizing recovery through perioperative nutritional status, promoting multimodal analgesia with a reduction in opioids, and early postoperative feeding [1,6]. In 1997, Kehlet suggested that while minor changes in perioperative care have no significant impact on their own, the additive value of multiple optimizations could drastically improve outcomes [6].

ERAS was implemented for the first time in colorectal surgery in the late 1990s and yielded reductions in postoperative hospital length of stay (mean difference [MD]: −1.64 days, 95% confidence interval [CI]: −2.21 to −1.08, *p* < 0.00001), postoperative complications (odds ratio [OR]: 0.57, 95% CI: 0.46 to 0.71, *p* < 0.00001), and readmission rates (OR: 0.57, 95% CI: 0.38 to 0.85, *p* = 0.006) and facilitated faster return of bowel function (MD: −0.74 days, 95% CI: −1.03 to −0.45, *p* < 0.00001) [7,8]. Ultimately, these results led to the ERAS study group publishing its first consensus guidelines for colorectal surgery in 2005 [6].

Following the initial success in colorectal surgery, ERAS principles were applied to other surgical disciplines, with each discipline tailoring protocols to suit its patient population and nuanced challenges [6]. For example, ERAS principles were adopted by orthopedic surgery in the early 2000s [6,8]. Orthopedic procedures, such as joint replacements and spinal surgeries, present distinct challenges, including an older patient population and higher levels of pain compared to colorectal surgery [9,10,11]. Despite these challenges, the implementation of ERAS protocols, such as prioritizing minimally invasive techniques, advanced blood management strategies, and early mobilization, resulted in a reduction in both hospital stay length and postoperative complications [9,10,11,12].

ERAS principles were also utilized in gynecologic surgeries in the early 2000s by employing enhanced nutritional support, structured postoperative care pathways, and early ambulation [11,12,13]. These strategies led to improvements in length of hospital stay, postoperative complications, cost, opioid use, and patient satisfaction [11,13]. Most recently, ERAS studies in urological surgery procedures via the use of protocol-driven bladder management have demonstrated a decline in postsurgical complications and accelerated bowel recovery [5,14].

### 3.2. Structure of ERAS in Non-Cardiac Surgery

Although the structure of ERAS varies from discipline to discipline and even surgery to surgery, ERAS protocols in non-cardiac surgery often consist of three distinct phases: preoperative, intraoperative, and postoperative [1]. These three phases align with the patient’s surgical journey from preparation for surgery through to recovery and discharge [3]. While each phase of the ERAS protocol independently contributes to improving patient care, the interplay of these components working in synchronicity is what truly drives expedited recovery following non-cardiac surgeries [1].

#### 3.2.1. Preoperative Phase

The preoperative phase of the ERAS protocol emphasizes strategies to minimize the physiological stress response, specifically through the optimization of the patient’s baseline health [1]. This includes risk stratifying and optimizing chronic conditions such as diabetes and hypertension, evaluation and management of organ dysfunction, alcohol abstinence, smoking cessation, reduction in fasting time, promoting the preoperative intake of liquid carbohydrates, micronutrition supplementation, and nutritional support [1]. Nutritional interventions such as carbohydrate loading instead of the traditional practice of overnight fasting, as well as smoking cessation, can reduce the risk of postoperative complications and specifically improve wound healing [1,15]. Moreover, preadmission counselling and patient-centred education of the postoperative care plan are key components of the preoperative phase as they empower the patient with knowledge about the surgical procedure and recovery to promote planning, while at the same time reducing anxiety [1].

#### 3.2.2. Intraoperative Phase

The intraoperative phase of the ERAS protocol places a large emphasis on anesthesia management and the ability to maintain optimal conditions throughout surgery in order to minimize stress and promote accelerated recovery [1]. Specifically, the use of regional anesthesia and goal-directed fluid therapy can minimize the stress response to surgery and maintain hemodynamic stability throughout the perioperative period, which, in turn, reduces postoperative complications [1]. Precise intravenous fluid management can also avoid complications such as volume overload and edema, therefore aiding in the promotion of early mobilization following surgery [1]. Other strategies, such as prophylactic antibiotics, minimally invasive surgical approaches, and minimizing surgical drains, nasogastric tubes, and urinary catheters, are utilized in this phase and can result in decreased pain, faster return of mobility, and decreased risk of postoperative infections [1].

#### 3.2.3. Postoperative Phase

The postoperative phase of the ERAS protocol is focused on early mobilization and oral intake. These goals are promoted by multimodal analgesia; less reliance on IV fluid therapy; thromboembolic and anti-emetic prophylaxis; hypoxemia and hypothermia prevention; removal of surgical drains, tubes, and catheters; uninterrupted chronic preoperative therapy; and careful follow-up post-discharge [1]. Prompt oral nutrition guided by the patient’s tolerance accelerates recovery through maintaining nutritional status and muscle strength [1], and early removal of drains, tubes, and catheters reduces the risk of infections and fosters mobility [1].

### 3.3. Variations of ERAS Among Different Non-Cardiac Surgeries

Although key features remain constant amongst ERAS protocols, there are several variations across disciplines as each ERAS protocol is often tailored to suit specific challenges and demands of each surgical subspecialty [1]. For example, colorectal surgery places significant emphasis on preoperative interventions like liquid carbohydrate loading and bowel preparation to maintain optimal nutrition while ensuring a clean surgical field during the procedure [1,7,8]. Intraoperative measures in colorectal surgery include the use of minimally invasive techniques to reduce postoperative pain and accelerate recovery [1,7,8]. In orthopedic surgeries, the preoperative strategies focus mainly on patient education to empower patients to participate actively in their recovery process and help manage patient expectations [9,10,12]. Moreover, as orthopedic procedures often involve blood loss, management of pre-existing conditions is especially important to minimize the need for blood transfusion during the surgery [1,9,10,12]. Gynecologic ERAS protocols prioritize enhanced multimodal pain management, including the use of regional nerve blocks to reduce opioid-induced nausea, and emphasize the adoption of minimally invasive laparoscopic techniques [1,11,14]. ERAS protocols in urological surgeries focus on structured protocols, including nutrition, to reduce the duration of urethral catheterization to prevent bladder-associated complications in this susceptible population [1,14].

### 3.4. Financial Outcomes of ERAS in Non-Cardiac Surgeries for Healthcare Systems

In addition to improving patient outcomes, ERAS protocols have resulted in significant financial benefits for the healthcare system. Through reducing the length of hospital stays, minimizing postoperative complications, and shortening recovery times, the implementation of ERAS protocols across various institutions has led to substantial cost reductions, allowing for improved resource allocation in the healthcare system [6,7,8,13,14,16,17,18]. ERAS implementation in colorectal, gynecologic oncology, urology, and pancreatic surgery has demonstrated decreased medication usage, reduced postoperative complications requiring follow-up, and decreased rates of revision procedures [12]. Together, these findings highlight the substantial financial advantages of integrating ERAS protocols into non-cardiac surgical practices, ultimately leading to better resource allocation and enhanced sustainability of the healthcare system.

### 3.5. Pitfalls and Challenges

One of the most significant and common challenges to implementing ERAS protocols is the resistance to change [19]. Securing multidisciplinary buy-in and coordinating consistent adherence to ERAS protocols across different departments and healthcare professionals can be difficult [13,14]. Another hurdle for the implementation of ERAS is the need for adequate infrastructure and resources. The effective implementation of ERAS requires investment in training time as well as specialized equipment and staff, which may strain the hospital budget [19]. While ERAS programs are generally cost-effective over time, the initial setup and ongoing costs associated with quality control and protocol updates may present as financial barriers to adoption [17]. Additional costs may also arise from the need to tailor and modify ERAS protocols to specific surgeries and patient groups, requiring ongoing research and adaptation to optimize outcomes [6]. Furthermore, patient education and compliance with preoperative and postoperative instructions are essential for successful ERAS implementation but can be challenging to achieve in practice [20]. Therefore, to overcome these challenges, a concerted effort from healthcare leaders, administrators, policymakers, and frontline medical staff is essential to ensure the successful integration and sustainability of ERAS protocols. Engaging these stakeholders through a collaborative approach will help integrate ERAS protocols more seamlessly, ultimately enhancing patient care and optimizing resource use [1].

## 4. ERAS in Cardiac Surgery and Cardiac Interventions

### 4.1. History of ERAS in Cardiac Surgery

Following their success in non-cardiac surgeries, ERAS principles were extended to cardiac surgery beginning in 2015 [21]. Formal Enhanced Recovery After Cardiac Surgery (ERACS) guidelines were subsequently developed and released in 2019 [2,22,23]. Following the release of these guidelines, Minacova et al. developed an ERACS pilot program involving 1981 cardiac procedures [5]. The program showed positive results, with patients being discharged as early as three days following coronary artery bypass grafting (CABG) and valve surgeries [5]. The project became an impetus for further research on ERACS, resulting in the publication of numerous randomized controlled trials and observational studies [24,25,26]. In a 2021 survey of 88 institutions in the United States, 9.1% had implemented an ERACS program [27], suggesting a slow and low adoption rate. The COVID-19 pandemic was a major contributor to this trend, with optimism that more centres would continue to adopt and implement ERACS in the post-pandemic era. In 2024, the ERAS Cardiac Society, ERAS International Society, and Society of Thoracic Surgeons issued a consensus statement updating the 2019 guidelines, with notable shifts toward patient-centred, multidisciplinary care [28]. As ERACS continues to evolve, there remains considerable opportunity for broader implementation across various centres and for its integration into the care of all cardiac surgery patients.

### 4.2. Elements of ERAS for Cardiac Surgery

Similar to ERAS for non-cardiac surgery, ERACS guidelines are organized into three phases: preoperative, intraoperative, and postoperative [4]. While these phases are distinct from one another, they work together to provide a holistic framework for patient care.

#### 4.2.1. Preoperative Phase

The hallmark of the preoperative phase is optimization of patient health indicators to reduce surgical risk [4]. A general recommendation is to conduct multifaceted screening to optimize patient outcomes [4]. For instance, screening for glycemic control, hypoalbuminemia, and anemia preoperatively can allow for optimization of the patient’s baseline health status [4]. For patients with hypoalbuminemia, oral nutritional supplementation is recommended seven to ten days before surgery when feasible [4]. Additionally, carbohydrate loading two hours prior to surgery is suggested to help regulate glucose levels [4]. One feature that distinguishes ERACS is the allowance of clear fluids up to 2–4 h before the administration of general anesthesia, based on established safety [4]. For elective surgeries, patients are also screened for smoking and alcohol consumption and are advised to abstain at least four weeks before surgery [4]. Additionally, providing thorough education about the procedure can help reduce preoperative anxiety [4]. To reduce frailty and immobility, enrolling patients in a cardiac prehabilitation program is recommended, offering support in areas such as nutrition, exercise, social support, education, and anxiety management [4].

#### 4.2.2. Intraoperative Phase

The recommendations for the intraoperative phase aim to reduce surgical risk. One of the main strategies to achieve this goal is the use of care bundles to prevent surgical site infections (SSIs) [4]. These care bundles, though relatively novel, are based on a combination of evidence-based interventions designed to reduce infection risk at multiple stages of the surgical process. Key components of the care bundle include topical intranasal therapies to reduce *Staphylococcus aureus* nasal carriage, weight-based cephalosporin infusion within 60 min before skin incision and re-infusion every 4 h to maintain effective prophylactic antibiotic levels throughout the procedure, and the use of meticulous skin preparation with antiseptics and routine dressing changes every 48 h to minimize microbial contamination and promote wound healing [4]. The success of these bundles depends on their comprehensive approach—when executed properly, evidence suggests that care bundles improve patient outcomes by standardizing practices, minimizing variability, and ensuring adherence to best practices, leading to a reduction in surgical infections [29].

For prevention of delirium, infection, and renal dysfunction, ERACS guidelines recommend avoidance of hyperthermia by maintaining normothermia through cardiopulmonary bypass rewarming combined with continuous surface rewarming [4]. Anesthetic management should also include protective lung ventilation strategies, with low tidal volumes during mechanical ventilation [4]. Rigid sternal fixation is advised for high-risk patients, such as those with a high body mass index or steroid use, to promote sternal healing [4]. Lastly, tranexamic acid or epsilon aminocaproic acid, with a maximum dose of 100 mg/kg, is recommended to reduce bleeding [4].

#### 4.2.3. Postoperative Phase

The postoperative guidelines are focused on pain control, early mobility, and early removal of lines/tubes/drains to promote healing. Like other disciplines, ERACS recommend a multimodal analgesic regimen combined with frequent pain assessments that are aligned with preoperative discussions with the patient [4]. Additionally, regular delirium screening is an important tenet of postoperative care [4]. To prevent postoperative hypothermia, strategies like forced air warming blankets, maintaining an increased room temperature, and using warm intravenous fluids are suggested [4]. To prevent surgical site infections, glycemic control with an insulin infusion is recommended as needed [4]. In the postoperative period, chest tubes are necessary to drain mediastinal blood; therefore, maintaining their patency is essential to prevent complications like tamponade or hemothorax. Active clearance of chest tubes has shown a reduction in postoperative atrial fibrillation rates [4]. According to the ERACS protocol, this patency should be preserved without breaking the sterile field, as doing so minimizes the need for additional interventions [4]. Furthermore, prophylactic anticoagulation should be initiated once homeostasis is achieved and continued until discharge to prevent vascular thrombotic events [4]. ERACS also recommends extubation within six hours of surgery, which is proven safe even for high-risk patients and helps reduce ICU stays and overall lengths of stay [4]. In addition to early extubation, early enteral feeding and mobilization are key elements of the ERACS protocol [4]. Monitoring urine biomarkers, such as tissue inhibitor of metalloproteinases-2 (TIMP-2) and insulin-like growth factor-binding protein 7 (IGFBP-7), for acute kidney injury in high-risk patients may help guide interventions [4]. Finally, goal-directed fluid therapy is advised to minimize postoperative complications. This fluid therapy includes balancing vasopressors, inotropes, crystalloid, and colloid fluids to avoid hypotension and low cardiac output, with blood pressure, cardiac index, systemic venous oxygen saturation, and urine output [4].

#### 4.2.4. 2024 Guideline Updates

It is important to underscore the inherent differences between cardiac and non-cardiac surgeries. In particular, these include nuances with respect to cardiovascular physiology and hemodynamic stability or instability. As such, the algorithms considered for ERAS and cardiac surgery should incorporate more complexities. While earlier work expanded on the foundation of ERAS in surgical specialties, emerging research in this area and major societal statements have strived to acknowledge the unique features associated with cardiac operations. The ERAS/STS 2024 joint consensus statement introduces several new recommendations [4,28]. For the preoperative phase, additional screenings for delirium risk factors, frailty, obstructive sleep apnea, and opioid tolerance are now recommended [28]. In the intraoperative phase, transesophageal echocardiography is recommended for those patients who are at high risk of perioperative morbidity or mortality [28]. For low-risk patients or procedures, the use of pulmonary artery catheters is discouraged as they do not improve outcomes and increase resource use [28]. Furthermore, for low-risk procedures, intraoperative or immediate postoperative extubation may be appropriate for some patients [28]. In the postoperative phase, the guidelines now include screening and prophylaxis for nausea and vomiting [28].

The 2024 statement also advises goal-directed fluid and hemodynamic therapy throughout the entire perioperative period, rather than limiting it to the postoperative phase [28]. In addition to the multimodal approach outlined in the 2019 guidelines, the new statement recommends chest wall regional anesthesia to manage perioperative pain [28]. Furthermore, for postoperative atrial fibrillation prevention, a multimodal approach is advised, including preoperative risk scoring, intravenous beta blockers or amiodarone administered preoperatively or immediately postoperatively, posterior pericardiectomy, and avoiding retained pericardial blood [28]. Another new perioperative recommendation is to use digital technologies, such as phone applications and wearable remote monitors, along with the engagement of multidisciplinary teams to better involve patients and their families in their care, optimizing the patient’s utilization of their healthcare network [29]. Finally, a recommendation is included for routine evaluation of the perioperative ERACS program to ensure high-quality care [29]. As a result of the new updates, the 2024 consensus statement provides a more patient-centred approach that considers the aging cardiac surgery population and builds on the evidence collected since the inception of ERACS to further refine and improve outcomes. A comparison of perioperative care elements recommended by the 2019 JAMA ERAS Cardiac guidelines and the 2024 ATS/STS ERAS Cardiac–STS Expert Consensus Document is provided in Table 1.

### 4.3. Successful Implementation of ERAS for Cardiac Surgery in an Adult Population

Since the development of ERACS guidelines in 2019, numerous studies have investigated the impact of ERACS on patient and system-level outcomes. One study compared 250 ERACS patients to 216 controls who underwent non-emergent cardiac surgery with median sternotomy, including ascending aorta repair, CABG, valve repair, and a combination of CABG and valve repair [30]. This found a 57% reduction in opioid use among ERACS patients from postoperative days 0–5 and earlier chest tube removal [30]. Among the various cardiac procedures, coronary artery bypass grafting (CABG) has been the most extensively studied, providing the strongest evidence base for ERACS implementation. An initial study implemented ERACS for 362 patients undergoing isolated, elective CABG surgery and compared them with 362 control patients [31]. The ERACS group experienced a 53.1% reduction in mechanical ventilation time, a 28.0% shorter ICU stay, and a 10.5% shorter hospital stay, along with significant reductions in complications like bronchopneumonia, acute respiratory distress syndrome, and severe acute kidney injury [31]. A subsequent study conducted by Takata et al. found a higher likelihood of early extubation in the ERACS group (46.0% vs. 35.8%) but no significant differences in 30-day morbidity or ICU stay in urgent and emergent CABG [27]. Beyond individual outcomes, ERACS in CABG has also demonstrated system-wide benefits. Sutton et al. analyzed over 1700 patients and found that ERACS implementation eliminated racial disparities in ICU readmission rates and hospital length of stay that were present in the control group [32]. These findings highlight that ERACS not only enhances clinical outcomes for CABG but also contributes to equitable care delivery across diverse patient populations.

**Figure 1:**
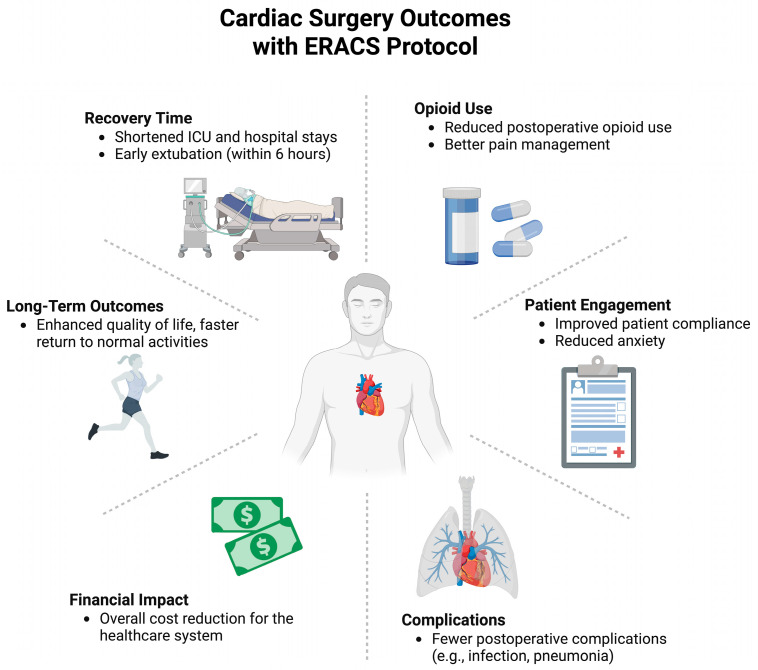
Key outcomes of cardiac surgery with the ERACS protocol, including improved recovery time, reduced opioid use, enhanced long-term outcomes, better patient engagement, reduced financial impact, and fewer complications.

Compared with CABG, fewer studies have evaluated ERACS specifically in valve surgery, but available evidence supports similar benefits. Jahangiri & Lamarche reported that optimized ERACS protocols enabled discharge as early as postoperative day 3 in selected valve surgery patients [5]. By avoiding chest wall trauma associated with sternotomy, mini-thoracotomy approaches, used for minimally invasive cardiac surgery (MICS), may further promote the advantages of ERACS. Although evidence for this is limited, reports do suggest that MICS patients following ERACS protocols have as good, and perhaps better, postoperative pain control, shorter IC and hospital stays, and reduced blood transfusions [33,34,35]. Zaouter et al., in an observational study including 23 patients, found ERACS protocol implementation decreased hospital length of stay by 3 days, with lower pain scores and trends toward global complication reduction [36]. This is further supported by a study by Petersen et al., which found an average reduction in hospital stay of 2 days following ERACS protocols for mini-AVR and mini-MVR surgeries for 61 patients [37]. Similarly, our group recently implemented a rapid-recovery protocol for minimally invasive mitral valve repair (MIMVR), in addition to standard ERACS protocols, showing that patients in the rapid-recovery group were able to achieve faster ICU and hospital discharge without an increase in complications or readmissions [33,38]. In a subpopulation analysis, most patients who underwent minimally invasive mitral valve surgery returned to baseline quality of life within two weeks, particularly in mobility and usual activities, highlighting the early benefits of ERAS protocols in minimally invasive procedures [33]. Therefore, as MICS continues to gain popularity, more data will emerge. There is potential that MICS may enhance the benefits of ERACS, with postoperative benefits of functional recovery and quality of life returning earlier than would be possible with ERACS alone. Results of the observational studies described are summarized in Table 2 and key outcomes of cardiac surgery with the ERACS protocol are displayed in Figure 1. 

### 4.4. Challenges of ERACS

While ERACS presents many benefits, the program also faces several challenges. The long-term costs and staffing requirements associated with ERACS are still unknown, especially if the program is applied broadly across all cardiac procedures at an institution rather than a specific subset. Another challenge is that many studies were conducted using versions of ERACS protocols before the official guidelines were published. Although these early protocols closely resemble the current guidelines, they do not fully reflect the standards currently recommended, making some of the existing data less applicable. While available data show short-term benefits and potential long-term advantages, additional duration of implementation is needed to validate these findings. Another stated limitation with ERACS is its reliance on a comprehensive program, which can be difficult in low-resource settings such as rural, low-income, or underserved areas [39]. Finally, while the ERACS guidelines provide a graded approach to the importance of each step, there is limited data on patient outcomes when only selected components are followed [4]. However, as ERACS is adopted in more centres and more trials are conducted, it is expected that tailored guidance will emerge for settings where full implementation is not feasible, allowing these centres to still benefit from the core principles of ERACS.

## 5. Perspective

The incorporation of ERAS protocols into cardiac surgery is a relatively recent advancement, with formal adoption only beginning with the publication of the ERAS Cardiac Surgery Guidelines in 2019 [4]. Prior to these guidelines, the implementation of ERAS principles varied widely among different institutions due to the absence of standardized approaches, resulting in considerable discrepancies in both practices and outcomes. However, over the past five years, the ERAS Cardiac Surgery Guidelines have expanded and are now more tailored to specifically reflect the complexities of cardiac procedures. During this time, there has also been an increase in the uptake of ERAS protocols in cardiac surgeries, suggesting a growing recognition of the potential benefits of ERAS protocols.

ERAS protocols in cardiac surgery face unique challenges and require targeted considerations compared to those in non-cardiac surgeries. One significant difference involves the complexity and risks associated with cardiac procedures and the use of cardiopulmonary bypass. As a result, cardiac surgery requires a heightened level of attention to perioperative cardiac function, meticulous hemodynamic monitoring, careful management of anticoagulation and its reversal, and precise fluid management. These measures are less emphasized in non-cardiac ERAS protocols, underscoring the need for tailored approaches to optimize outcomes in cardiac surgery.

One of the previously identified key barriers to successful implementation of ERACS protocols is a lack of frontline provider buy-in [40]. Providers may be hesitant to adopt new protocols due to unfamiliarity with protocol components or skepticism regarding the evidence or necessity of their use [40]. The creation of turn-key order sets by the ERACS working group has aimed to alleviate some of these difficulties in transition [41,42,43,44,45]. These order sets may help ease some of the workload and administrative burden of establishing ERACS in institutions. Having premade order sets may also allow for reductions in variation in care and simplify clinician workflows with defined cognitive aids, guiding their clinical practice. Ultimately, this barrier highlights the need for routine auditing of ERACS implementation and continuous feedback to adapt protocols to each individual site.

Moreover, while ERAS guidelines for cardiac surgery are comprehensive, there could be additional benefits from integrating additional elements of existing ERAS protocols used in non-cardiac surgeries. For instance, in colorectal and gynecologic surgeries, the administration of alvimopan during the preoperative period is encouraged to reduce postoperative ileus [46]. Given that many cardiac surgery patients experience preoperative constipation, which can exacerbate postoperative gastrointestinal issues like ileus [36,47], introducing agents such as alvimopan or laxatives or promoting a high-fibre diet could help mitigate these complications. During the intraoperative period, non-cardiac ERAS protocols often recommend goal-directed fluid therapy to prevent fluid overload [1]. In contrast, current cardiac ERAS guidelines do not provide specific intraoperative fluid management recommendations, despite its importance in stabilizing hemodynamics, preventing complications such as fluid overload and kidney dysfunction, optimizing cardiac and organ perfusion, and supporting recovery in cardiac surgeries [1,48]. Incorporating fluid management strategies from non-cardiac ERAS protocols could help reduce fluid-related complications in cardiac surgery patients [1,48]. Furthermore, non-cardiac ERAS guidelines emphasize early ambulation to lower the risk of pneumonia and venous thromboembolism [49,50]. Given the prolonged recovery times often seen in cardiac surgery patients, encouraging early mobilization could be crucial for preventing these complications and promoting quicker recovery. Finally, minimally invasive surgical techniques are a component of non-cardiac ERAS guidelines [7,11,51]. With the recent popularization of minimally invasive surgeries in cardiac surgery, further study into both the effectiveness of ERAS in this surgical population and the impact of minimally invasive techniques as a component of traditional cardiac surgery could be explored.

Currently, many of the results of ERACS quality improvement studies involve hospital stay as a measurement of the success of a protocol, with various unstandardized measures of morbidity and mortality [52]. This lack of standardization in outcome measurement means clear consensus on the efficacy of ERACS protocols in the literature is difficult to ascertain. An expert consensus by Hirji et al. identified 21 data points for adherence [52]. Standardized scores such as the Clavien–Dindo classification scores popularized in general surgery ERAS could be adapted to cardiac surgery to aid in standardization and quality improvement measures [53]. The Clavien–Dindo Classification is a method of categorizing procedures based on the severity of the resulting surgical complications, ranging from grade I complications—those that do not require pharmacologic, surgical, endoscopic, or radiological interventions—to grade V complications, including patient death [53]. Furthermore, addition of standardized patient-centred outcomes such as time to return to work and hospital readmission rates would help focus protocols and measurements to the primary aim of ERACS protocols, patient care. Ultimately, consensus on goals and measured outcomes would likely improve the ability to tailor recommendations in the future.

Given that protocols will continue to evolve, there will likely be a focus on refining the protocols based on emerging studies, incorporating newer technologies, and attempting to bridge existing knowledge gaps, such as the effectiveness of ERAS in minimally invasive cardiac surgeries. Future research should be aimed at standardizing ERAS protocols across institutions, including comprehensive systematic reviews to clarify the heterogeneity of outcomes, and ideally, large-scale randomized control trials. Lastly, studying patient-reported outcomes and quality of life measures could provide a more comprehensive and holistic understanding of the impact of ERAS in cardiac surgery.

It is also important to make note of the potential role ERAS protocols can have in patients who require mechanical circulatory support (MCS). While data is sparse in this area, with the rapid increase in use of various types of MCS devices, groups and centres should consider developing ERAS protocols that can be dedicated to temporary and durable MCS patients. The PeriOperative Quality Initiative (POQI) and ERACS working groups have recently published a statement that can be used as a framework for centres with MCS programs who wish to implement ERAS protocols [54]. Evaluating the efficacy and impact of these protocols will likely take a while, and standardizing them across sites and jurisdictions will not be trivial. However, given the overall benefits of ERAS in other cardiac surgical patients, there should be optimism in the potential advantages of ERAS in MCS patients.

Finally, given the rapid evolution of artificial intelligence (AI) and its burgeoning application to different areas of Medicine, ERAS and ERACS stakeholders should play a central role in assessing the potential role AI can have in developing, improving, and implementing ERAS protocols. Recently, a Dutch group validated machine learning concepts in gastrointestinal and oncology surgery patients to predict safe patient discharge [55]. The study underscored how big data, machine learning, and AI can be integrated into clinical workflow and expedite patient discharge. As our understanding of AI in medicine improves, cautious but concrete steps can be taken to wield the enormous potential of AI to improve patient experience and outcomes.

## 6. Conclusions

Although ERAS protocols have demonstrated benefits in non-cardiac surgeries, implementation within cardiac surgery continues to be a work in progress. Indeed, the evolution of ERACS guidelines, based on a growing body of the dedicated literature, suggests that more centres may be compelled to consider adopting ERACS protocols. As the field continues to evolve, knowledge gaps can be addressed through rigorous research; innovative strategies can be designed and evaluated; and outcome measures can be better defined and standardized. While the evidence to date demonstrates encouraging outcomes with ERACS implementation, interpretation of results is limited by variability in protocols, heterogeneity of outcome reporting, and reliance on observational data. Future research, including standardized multicenter randomized trials, is needed to strengthen the evidence base and guide consistent implementation across cardiac surgical populations. The ultimate goal is to improve patient experience and outcomes, and based on the current literature evaluated, the advancement of ERACS protocols should continue to facilitate these important objectives.

## Figures and Tables

**Table 1:** Comparison of perioperative care elements recommended by the 2019 JAMA ERAS Cardiac guidelines and the 2024 ATS/STS ERAS Cardiac–STS Expert Consensus Document. Elements are colour-coded by level of evidence: High (green), Moderate (yellow), Low (orange), and Harm/No Benefit (red). Abbreviations: AKI, acute kidney injury; ATS, *Annals of Thoracic Surgery*; CNS, central nervous system; CPB, cardiopulmonary bypass; COR, Class of Recommendation; ERAS, Enhanced Recovery After Surgery; JAMA, *Journal of the American Medical Association*; LOE, level of evidence; NPO, nil per os; NGT, nasogastric tube; PA, pulmonary artery; PONV, postoperative nausea and vomiting; TEE, transesophageal echocardiography.

Care Element	JAMA 2019	ATS/STS 2024
Auditing of process measures	Not Included	Moderate
Avoidance of hypothermia <36 °C after CPB	I; COR B-NR (Moderate)	Covered intraop (Moderate)
Avoiding PA catheters in low-risk patients	Not Included	Moderate
Carbohydrate loading	IIb; COR C-LD (Low)	Covered under NPO/Prehab (Low)
Chemical/mechanical thromboprophylaxis	IIa; COR C-LD (Low)	Covered under screening (Moderate)
Chest tube patency	I; COR B-NR (Moderate)	Not Included
Chest wall regional analgesia	Not Included	Moderate
Clear liquids up to 2–4 h preop	IIb; COR C-LD (Low)	Low
Comprehensive patient blood management	Not Included	Moderate
Continuing ventilation on CPB	Not Included	Moderate
Correction of nutritional deficiency	IIa; COR C-LD (Low)	Not Included
Early ambulation and upper extremity exercise	Not Included	Moderate
Establishment of multidisciplinary team	Not Included	Moderate
Facilitate extubation within 6 hrs	IIa; COR B-R (Moderate)	Moderate
Goal-directed fluid and hemodynamic therapy	I; COR B-R (High)	Moderate
Goal-directed perfusion while on CPB	Not Included	Low
Hyperthermia >37.9 °C rewarming CPB	III Harm	N/A
Insulin infusion for hyperglycemia	IIa; COR B-NR (Moderate)	Covered under screening (Moderate)
Limiting NPO status for clear fluids >2 h	IIb; COR C-LD (Low)	Low
Mechanical ventilation with lung protective strategies	Not Included	High
Multicomponent prehabilitation	IIa; COR B-R (Low)	Low
Multifaceted screening and risk assessment	Not Included	Moderate
Multimodal analgesia with opioid stewardship	I; COR B-NR (Moderate)	Moderate
Multimodal pain plan	I; COR B-NR (Moderate)	Moderate
Patient engagement through shared decision-making	IIa; COR C-LD (Low)	Low
Patient engagement tools/education	IIa; COR C-LD (Low)	Low
Perioperative glycemic control	I; COR B-R (High)	Covered under screening (Moderate)
Preop HbA1c measurement	IIa; COR C-LD (Low)	Covered under screening (Moderate)
Prevention of POAF	Not Included	Moderate
Rigid sternal fixation for high-risk	IIa; COR B-R (Moderate)	Not Included
Risk assessment/prophylaxis for PONV	Not Included	Moderate
Routine delirium screening and nonpharm tx	I; COR B-NR (Moderate)	High
Screening and AKI care model	IIa; COR B-R (Moderate)	Moderate
Selective intra/post-op extubation in low-risk	Not Included	Low
Smoking and alcohol cessation ≥4 wks preop	I; COR C-LD (Low)	Covered under screening (Low)
Standard use of CNS monitoring	Not Included	Moderate
Stripping/breaking sterile chest tubes	III No Benefit	N/A
Surgical site infection reduction bundle	I; COR B-R (High)	High
TEE in moderate-/high-risk patients	Not Included	Moderate
Tranexamic acid for on-pump surgery	I; COR A (High)	Not Included

**Table 2:** Summary of observational studies comparing outcomes in patients who received surgeries with Enhanced Recovery After Cardiac Surgery with control patients as described in Section 4.3. Abbreviations: CABG, coronary artery bypass surgery; ERACS, Enhanced Recovery After Cardiac Surgery; ICU, intensive care unit.

Reference Number	Study Title	Year	Number of ERACS Patients	Number of Control Patients	Intervention	Results of ERACS Implementation
[26]	Enhanced Recovery After Surgery Is Associated with Reduced Hospital Length of Stay after Urgent or Emergency Isolated Coronary Artery Bypass Surgery at an Urban, Tertiary Care Teaching Hospital: An Interrupted Time Series Analysis with Propensity Score Matching	2023	565	330	Isolated Coronary Artery Bypass Surgery	Higher likelihood of early extubation (46.0% vs. 35.8%) No significant differences in 30-day morbidity or ICU stay in urgent and emergent CABG
[30]	Enhanced recovery after cardiac surgery protocol reduces perioperative opioid use	2022	250	216	Non-emergent cardiac surgery with median sternotomy (including ascending aorta repair, CABG, valve repair, and a combination of CABG and valve repair)	57% reduction in opioid use from postoperative days 0–5 Earlier chest tube removal ICU and hospital length of stay and 30-day morbidity and mortality showed no significant differences
[31]	Enhanced recovery after surgery program for patients undergoing isolated elective coronary artery bypass surgery improves postoperative outcomes	2023	362	362	Isolated, elective CABG surgery	53.1% reduction in mechanical ventilation time 28.0% shorter ICU stay 10.5% shorter hospital stay Significant reductions in complications like bronchopneumonia, acute respiratory distress syndrome, and severe acute kidney injury
[32]	Enhanced Recovery After Surgery Is Associated With Improved Outcomes and Reduced Racial and Ethnic Disparities After Isolated Coronary Artery Bypass Surgery: A Retrospective Analysis With Propensity-Score Matching.	2022	1079	656	Isolated Coronary Artery Bypass Surgery	Eliminated racial disparities in postoperative ICU readmission and length of stay
[36]	Reduced Length of Hospital Stay for Cardiac Surgery-Implementing an Optimized Perioperative Pathway: Prospective Evaluation of an Enhanced Recovery After Surgery Program Designed for Mini-Invasive Aortic Valve Replacement.	2019	23	23	Mini-Invasive Aortic Valve Replacement	Decreased hospital length of stay by 3 days Lower pain scores, Trends toward global complication reduction
[37]	Economic impact of enhanced recovery after surgery protocol in minimally invasive cardiac surgery.	2021	61	69	Elective minimally invasive aortic or mitral valve surgery	Average reduction in hospital stay by 2 days

## Data Availability

The original contributions presented in this study are included in the article. Further inquiries can be directed to the corresponding author(s).
